# Public Beliefs on Antibiotics and Symptoms of Respiratory Tract Infections among Rural and Urban Population in Poland: A Questionnaire Study

**DOI:** 10.1371/journal.pone.0109248

**Published:** 2014-10-02

**Authors:** Maciek Godycki-Cwirko, Jochen W. L. Cals, Nick Francis, Theo Verheij, Christopher C. Butler, Herman Goossens, Izabela Zakowska, Lech Panasiuk

**Affiliations:** 1 Centre for Family and Community Medicine, Medical University of Lodz, Lodz, Poland; 2 Department of Family Medicine, CAPHRI School for Public Health and Primary Care, Maastricht University, Maastricht, The Netherlands; 3 Cochrane Institute of Primary Care and Public Health, School of Medicine, Cardiff University, Cardiff, United Kingdom; 4 Julius Center for Health Sciences and Primary Care, University Medical Center, Utrecht, The Netherlands; 5 Cochrane Institute of Primary Care and Public Health, School of Medicine, Cardiff University, Cardiff, United Kingdom; Department of Primary Care Health Sciences, University of Oxford, Oxford, United Kingdom; 6 Laboratory of Medical Microbiology, Vaccine & Infectious Disease Institute (VAXINFECTIO), University of Antwerp, Antwerp, Belgium; 7 Department of Family Medicine, Institute of Rural Health, Lublin, Chair and Department of Family Medicine, Medical University, Lublin, Poland; University of Economics and Innovation, Lublin, Poland; Alberta Provincial Laboratory for Public Health/University of Alberta, Canada

## Abstract

**Introduction:**

General public views and expectations around the use of antibiotics can influence general practitioners' antibiotic prescribing decisions. We set out to describe the knowledge, attitudes and beliefs about the use of antibiotics for respiratory tract infections in adults in Poland, and explore differences according to where people live in an urban-rural continuum.

**Material and Methods:**

Face to face survey among a stratified random sample of adults from the general population.

**Results:**

1,210 adults completed the questionnaire (87% response rate); 44.3% were rural; 57.9% were women. 49.4% of rural respondents and 44.4% of urban respondents had used an antibiotic in the last 2 years. Rural participants were less likely to agree with the statement “usually I know when I need an antibiotic,” (53.5% vs. 61.3% respectively; p = 0.015) and reported that they would consult with a physician for a cough with yellow/green phlegm (69.2% vs. 74.9% respectively; p = 0.004), and were more likely to state that they would leave the decision about antibiotic prescribing to their doctor (87.5% vs. 85.6% respectively; p = 0.026). However, rural participants were more likely to believe that antibiotics accelerate recovery from sore throat (45.7% vs. 37.1% respectively; p = 0.017). Use of antibiotic in the last 2 years, level of education, number of children and awareness of the problem of developing antimicrobial resistance predicted accurate knowledge about antibiotic effectiveness.

**Conclusions:**

There were no major differences in beliefs about antibiotics between urban and rural responders, although rural responders were slightly less confident in their knowledge about antibiotics and self-reported greater use of antibiotics. Despite differences in the level of education between rural and urban responders, there were no significant differences in their knowledge about antibiotic effectiveness.

## Introduction

Public knowledge, attitude and beliefs can influence physicians' decisions to prescribe antibiotics for respiratory tract infections (RTI) [1], [2], [3], [4], [5]. Patient expectations have been found to strongly predict antibiotic prescribing, and are mediated by patients' knowledge, beliefs, and experiences of antibiotics [6]. Greater knowledge amongst patients about the appropriate indications for antibiotic therapy has been associated with lower antibiotic prescription rates [7]. Patients' expectations for antibiotics and physicians' assumptions regarding these expectations are associated with antibiotic prescribing for RTIs [2], [8], with patients who are perceived by physicians as expecting an antibiotic being 10 times more likely to be prescribed one [10]. Physicians describe prescribing in order to not jeopardize relationships with their patients [9]. However, patients' views are not always congruent with bio-medical views about the appropriate use of antibiotics.

Understanding public knowledge, attitudes and beliefs about antibiotics can be of value in helping to design appropriate interventions, but not much is known about the views of certain subpopulations. Antibiotic prescribing for RTIs varies between countries [11], but no study has yet investigated variation in antibiotic prescribing and public views about antibiotic use between urban and rural residents within a country. According to the World Organization of Family Doctors there are special problems in rural health and research is needed to inform rural health initiatives and to monitor progress in rural health care [12]. A study of rural parent behaviours and expectations when caring for children with acute RTIs found that awareness of the help seeking behaviours and expectations of patients in rural communities would allow health care professionals to provide care more effectively [13]. Physicians practicing in rural settings have been shown to prescribe antimicrobials more frequently compared with those in urban settings (83.8%, and 68.2% respectively) [14].

Polish GPs have been shown to be amongst the highest antibiotic prescribers for RTIs in Europe [15], [16], [17] and 40% of the Polish population lives in rural areas. This study aimed to explore differences between the rural and urban adult populations in Poland in terms of antibiotic use for RTIs and knowledge, attitudes and beliefs about antibiotic use for RTI symptoms.

## Material and Methods

### Survey

Members of the Polish adult (aged 18–89 years) general population were surveyed as part of an omnibus survey (http://obop-arch.tnsglobal.pl/omnimas) in which respondents were asked to address social, political and consumer topics. The survey was conducted between 6th and 11th May 2011 by a Polish company (TNS OBOP http://www.obop.com.pl) using a random route sampling technique. First, a sample of 1210 groups of people living together in a household were randomly selected from all Polish households. Then an interviewer visited the households and invited the adult person who most recently had a birthday to participate in an interview. If this person was absent then a new visit date was scheduled (in the next 2–4 days) or if it was impossible then adult with the next most recent birthday was selected (from the same household).

Data was collected through face-to-face interviews and Computer Assisted Personal Interviews (CAPI). Every respondent was informed about the study and provided verbal consent, which was documented as an answer to initial survey question.

The sample was representative for the Polish population stratified for gender, age, education level, region (voivodeship) and the number of inhabitants living in the area. The region in which respondents lived were formally classified as rural or urban. Rural areas included villages defined as units of compact settlement or dispersed settlements with existing functions of agricultural or related services or tourism, having no rights or status as a town, nor local community facilities such as a town hall or public offices. A town was defined as a settlement having mostly concentrated housing and non-agricultural functions, possessing town rights or the status of a town pursuant to provisions of separate laws [18], [19].

Interviewers asked the responders eight questions with sub items taken from a computer-based questionnaire, and our analysis followed the approached of Cals and colleagues [23].

Baseline questions inquired about respondents' perceptions of their own health status, whether they had taken any antibiotics in the last 2 years and if so where they got the antibiotics from, and what their main source of knowledge about antibiotics was. They were then asked questions relating to their knowledge about the effects of antimicrobials, their beliefs on the effectiveness of antibiotic in resolving symptoms for nine RTIs, their attitude towards antibiotic use, and whether they thought it was necessary to see a physician for nine respiratory symptoms using a 5-item Likert scale with response ranging from “strongly disagree” to “strongly agree”. They were also asked if they considered antibiotics to be effective against bacteria and/or viruses, and were given the option or responding “I do not know” in addition to “yes” and “no”.

### Ethics Statement

The survey was presented to an institutional review board (TNS Review Group), who decided that ethical approval was not required. Ethical approval is only required in Poland only if sensitive personal data are used or if the study involves the administration of an active substance or therapy. Participants were aware that their responses would be analyzed to establish the group opinion on the topics they were asked about and they agreed to participate in the survey. Our study was anonymised and no personal sensitive data were collected.

### Statistics

Data were analysed with STATISTICA 10 for PC (Statsoft, Inc.) and presented as the crude number and proportion (percentage) of responses. Most questionnaires were fully completed. Missing data were excluded from the analysis and cross tables of pre-selected variables for total responders, rural and urban areas were calculated. Variables were coded into the following categories: education according to the number of years of education (low - at least 9, medium - at least 12 and high - at least 15); economic status (good, medium and poor as rate by respondents); health status (good, poor, not known [“I don't know”]; as rated by respondents); sources of information on antibiotics (health care system, mass media, private and “I don't know”). To identify variables associated with setting (rural vs. urban) χ^2^ tests were performed. The normality data distribution was examined using Shapiro-Wilk test. Differences between different groups were tested using the Mann-Whitney U test for data with not normal distribution. The results were presented as median (Me), interquartile range (IQR), range (min, max) or n (%). P values of 0.05 or less were considered to be statistically significant.

To identify factors related to knowledge about antibiotic effectiveness we used logistic regression models for binary responses. The significance of the variables in the model were assessed by the Wald χ^2^ test and confidence intervals. The fit of the model was assessed by the goodness of fit χ^2^ test. To assess outliers and detect extreme points in the design space, logistic regression diagnostics were performed by plotting the diagnostic statistics deviance residuals analyses. For the multiple logistic regression model variables with p≤0.10 were selected. Odds ratios (ORs) with corresponding 95% confidence intervals (CIs) were calculated.

## Results

### Demographic data

We received 1210 questionnaires (87% response rate), with 44.3% from rural responders. 57.9% of responders were women, with 56.0% of these coming from rural areas. The median age for responders was 49 years (interquartile range [IQR], 34, 61 years). The median age of responders in rural and urban areas were 48.0 years ([IQR], (34, 61)) and 50.0 years ([IQR], (33, 62)) respectively (p = 0.340).

46.1% of responders had a low level of education, 37.4% medium and 17.2% high. There was significant difference in level of education between rural and urban areas: low - 56.7% in rural vs. 37.0% in urban; medium - 32.8% vs. 40.5% respectively; and high -10.5% vs. 22.6% respectively (p<0.01).

Only 20.5% perceived their economic status as good [16.6% in rural areas vs. 23.6% in urban ones (p = 0.00001)]. Single (including divorced and widowed) marital status was more common in urban areas (46.29%) compared to rural areas (31.53%) (p<0,0001).

### Health status and antibiotics' use

74.7% of respondents perceived their health as good (73% in rural vs. 76% in urban areas), and 47% reported having used an antibiotic within the past 2 years (50% of rural responders vs. 44% urban [p = 0.038]). In both settings antibiotics were almost exclusively obtained via a prescription for themselves from a physician (97% in rural and 98% in urban area).

### Knowledge about antibiotics

26.4% of respondents indicated that they thought that antibiotics were effective against bacteria and not viruses, the bio-medically correct answer. There was no significant difference by area (26.5%% in rural vs. 26.3%% in urban; p = 0.928). 71.7% indicated that their knowledge about antibiotics came primarily from their physicians and medical centres. There was no significant difference by area (73.32% in rural and 70.33% in urban; p = 0.314). Other reported sources of information for all responders were: mass media (TV, radio, newspapers, internet and public campaigns) in 12.4% and from individuals, such as family members, friends or neighbours in 8.9%.

### Attitude towards antibiotic use and physicians assistance for RTIs' symptoms

53% of respondents from rural areas and 61% from urban areas (p = 0.015) indicated that they usually knew when they needed antibiotics. The majority of respondents indicated that they would leave the decision to prescribe to their doctor (Table 1). Table 2 shows the number of respondents who would consult a GP with various symptoms. No important differences between rural and urban respondents were found.

**Table 1:** Attitudes towards antibiotics for RTI symptoms*.

Attitude	Responders n(%)	Rural n(%)	Urban n(%)
Usually I know when I need antibiotics	700 (57.9)	287 (53.5)	413 (61.3)**
Decision of using antibiotic better leave to the doctor	1046 (86,5)	469 (87.5)	577 (85.6)**
I can buy a prescribed antibiotic by your doctor, and take it when the symptoms getting worse take it with delay	449 (37.1)	199 (37.1)	250 (37.1)
Bacteria can become more resistant to antibiotics	932 (77)	411 (76.7)	521 (77.3)**
Bacteria resistant to antibiotics are present only in hospitals	389 (32.2)	164 (30.6)	225 (33.4)
If antibiotics were used before, they will be again required for similar symptoms	716 (59.2)	323 (60.3)	393 (58.3)

* - answers “I partially agree” or “I strongly agree” were included.

**- P<0.05 for living area comparison.

**Table 2:** Condition specific rates of respondents with belief about the need for a GP consultation *.

Symptom	Responders n(%)	Rural n(%)	Urban n(%)
Sore throat	376 (31.1)	167 (31.2)	209 (31)
Cough with transparent phlegm	673 (55.6)	299 (55.8)	374 (55.5)
Cough with yellow/green phlegm	876 (72.4)	371 (69.2)	505 (74.9)**
Cough with fever	879 (72.6)	389 (72.6)	490 (72.7)
Cough lasting more than 2 weeks	980 (81)	428 (79.9)	552 (81.9)
Respiratory tract infection	830 (68.6)	359 (67)	471 (69.9)
Common cold	395 (32.6)	167 (31.2)	228 (33.8)
Acute bronchitis	1125 (93)	495 (92.4)	630 (93.5)
Pneumonia	1140 (94.2)	503 (93.8)	637 (94.5)

*- answers “I need the doctor's help” or “strongly believe I need the doctor's help” were included.

**- P<0.05 for living area comparison.

### Beliefs about use of antibiotics for RTIs' symptoms

Respondents living in rural areas more often believed that antibiotics improved resolution of symptoms such as sore throat (45.7% and 37.1% for rural and urban respectively; p = 0.017), cough with clear phlegm (56.2% and 48.8% for rural and urban respectively; p = 0.044), cough with yellow/green phlegm (67.2% and 64.8% for rural and urban respectively; p = 0.022) and cough with fever (70.2% and 61.7% for rural and urban respectively; p = 0.011). 38.9% of all responders believed that antibiotics improved common cold symptoms (42.4% rural vs. 36.2% of urban ones; p = 0.147). 87.5% of responders (87.9% rural vs. 87.2% urban; p = 0.788) believed that antibiotics aided recovery in acute bronchitis and 90.5% (90.5% rural vs. 90.5% urban; p = 0.089) in pneumonia (Table 3)

**Table 3:** Condition specific rates of respondents who believe that antibiotics can accelerate recovery*.

Symptoms	Responders n(%)	Rural n(%)	Urban n(%)
Sore throat	495 (40.91)	245 (45.7)	250 (37.1)**
Cough with transparent phlegm	630 (52.1)	301 (56.2)	329 (48.8)**
Cough with yellow/green phlegm	797 (65.9)	360 (67.2)	437 (64.8)**
Cough with fever	792 (65.5)	376 (70.2)	416 (61.7)**
Cough lasting more than 2 weeks	823 (68)	371 (69.2)	452 (67.1)**
Respiratory tract infection	807 (66.7)	370 (69)	437 (64.8)
Common cold	471 (38.9)	227 (42.4)	244 (36.2)
Acute bronchitis	1059 (87.5)	471 (87.9)	588 (87.2)
Pneumonia	1095 (90.5)	485 (90.5)	610 (90.5)

*- answers “antibiotics rather accelerate healing” or “antibiotics accelerate healing” were included.

**- P<0.05 for living area comparison.

### Predictors of accurate knowledge about antibiotic effectiveness

Recognition of the problem of developing antimicrobial resistance (OR 2.87, 95% CI = 1.88–4.40), high level of education (OR 3.19, 95% CI = 2.13–4.76) and being a parent (having two or more children aged ≤12 years) (OR 3.40 95% CI = 2.20–5.26) were the strongest predictors of accurate knowledge of antibiotic effectiveness (Table 3). Awareness of the problem of antimicrobial resistance was a stronger predictor of knowledge about antibiotic effectiveness in rural (OR = 5.23, 95% CI = 2.27–12.06) compared to urban (OR = 2.23, 95% CI = 1.35–3.68) areas. Use of antibiotics in the last 2 years was not a predictor of accurate knowledge in the rural subsample (Table 4).

**Table 4:** Factors independently associated with accurate knowledge of antibiotic effectiveness.

Factor	Responders OR	Rural OR	Urban OR
Use of antibiotic in the last 2 years	1.42* 1.40*	–	1.53*
Level of education	1.75*	1.60*	1.86*
Low	1.00 (ref.)		
Medium	1.68*		
High	3.19*		
No. of children	1.66*	1.59*	1.67*
None	1.00 (ref.)		
One			
Two	3.40*		
Three and more	2.57*		
Acknowledgement of developing antimicrobial resistance	2.85* 2.87*	5.23*	2.23*

*- P<0.05.

## Discussion

The purpose of this study was to explore differences in perceptions of antibiotic use for RTIs between people living in rural and urban areas. We found no important differences between rural and urban populations. More rural responders had used an antibiotic during the previous 2 years, which possibly reflects a greater concern about their health and a belief in the need for prompt action when they develop symptoms.

Despite statistically significant differences in the level of education between respondents living in rural and urban areas, we found no statistically significant differences in accurate knowledge about antibiotic effectiveness between these groups. This could be because GPs and their practices are the main source for patients' knowledge on antibiotics, and GPs might also address beliefs about antibiotics during consultations for RTIs. Physicians' decisions may be challenged by patients who have perceptions about the effectiveness of antibiotics that do not conform with the biomedical viewpoint. A pan-European survey showed that 53% of Europeans still believed that antibiotics kill viruses (http://ec.europa.eu/health/antimicrobial_resistance/docs/ebs_338_en.pdf. 2010).

A US study investigating public views on antibiotics found that 55% patients incorrectly identified antibiotics as being effective against viruses [20]. Other studies have also found that a large proportion of people believe that antibiotics improve outcomes for bacterial as well as viral respiratory infections [20], [21], [22]. There are also public misconceptions on the effectiveness of, and indications for antibiotics [23].

In a Dutch study 44.6% of respondents correctly identified that antibiotics are effective against bacteria and not viral infections [23], which is slightly higher than was found in our population. Half of our respondents endorsed the incorrect belief that antibiotics are effective against infections caused by viruses. Only 1 in 4 respondents correctly endorsed both of the following statements: (1) that antibiotics are effective against bacterial and (2) antibiotics are ineffective against viral infections. There were no significant differences between rural and urban respondents in their knowledge about the effectiveness of antibiotics in treating RTIs such as the common cold, acute bronchitis, or pneumonia, or infections caused by bacteria. There were also no differences between rural and urban respondents with respect to belief in the need for help from a physician for all symptoms, except cough with yellow/green phlegm and a cough with fever. Rural patients were less likely to indicate that they would seek medical attention for these two symptoms. Most of the respondents, especially in rural areas, agreed that the decision to use antibiotics should be taken by a physician.

There were high levels of agreement that antibiotics improve recovery in pneumonia. However one in 10 respondents indicated that they wouldn't rely on antibiotics for pneumonia. An interesting finding was that there were high levels of agreement that antibiotics improve recovery in bronchitis, suggesting that responders might not differentiate it from pneumonia. Compared to Dutch respondents Polish respondents, irrespective of setting, were more likely to indicate that antibiotics are generally effective for a cough lasting two weeks [23].

Education level, having used an antibiotic in the last 2 years, and having more than 1 child were all associated with appropriate knowledge about antibiotic effectiveness. Education levels were also associated with correct beliefs in the Netherlands. Having more children increases the chance of consulting and therefore the opportunity for discussions with health carers about the appropriate use of antibiotics.

### Strengths and limitations

This is the first study comparing the views of rural vs. urban respondents regarding antibiotics and respiratory tract infections. This was a relatively large study of randomly selected adults in the community and had a high response rate.

As in every survey, selection bias is a possibility. The survey was conducted in May when individuals are less likely to experience RTIs and might have had difficulty accurately recalling infection-related behaviours.

## Conclusions

- We found evidence that specific patient factors, such as antimicrobial awareness, education and number of children, are associated with accurate knowledge about antibiotic effectiveness.
- Residence in a rural or urban setting was not associated with differences in patient factors, although rural responders self-reported more use of antibiotics for RTIs, and had slightly less confidence in their knowledge about antibiotics and greater belief in their effectiveness.
- Despite significant differences between rural and urban levels of education, there were no statistically significant differences in rural and urban knowledge about the effectiveness of antibiotics for bacterial and viral infections.
